# The Functional Significance of MicroRNA-29c in Patients with Colorectal Cancer: A Potential Circulating Biomarker for Predicting Early Relapse

**DOI:** 10.1371/journal.pone.0066842

**Published:** 2013-06-28

**Authors:** I-Ping Yang, Hsiang-Lin Tsai, Ching-Wen Huang, Ming-Yii Huang, Ming-Feng Hou, Suh-Hang Hank Juo, Jaw-Yuan Wang

**Affiliations:** 1 Department of Medical Genetics, College of Medicine, Kaohsiung Medical University, Kaohsiung, Taiwan; 2 Department of Nursing, Shu-Zen Junior College of Medicine and Management, Kaohsiung, Taiwan; 3 Division of General Surgery Medicine, Department of Surgery, Kaohsiung Medical University Hospital, Kaohsiung, Taiwan; 4 Cancer Center, Kaohsiung Medical University Hospital, Kaohsiung Medical University, Kaohsiung, Taiwan; 5 Graduate Institute of Medicine, College of Medicine, Kaohsiung Medical University, Kaohsiung, Taiwan; 6 Division of Gastrointestinal and General Surgery, Department of Surgery, Kaohsiung Medical University Hospital, Kaohsiung Medical University, Kaohsiung, Taiwan; 7 Department of Surgery, Kaohsiung Municipal Hsiao-Kang Hospital, Kaohsiung Medical University, Kaohsiung, Taiwan; 8 Department of Radiation Oncology, Faculty of Medicine, College of Medicine, Kaohsiung Medical University, Kaohsiung, Taiwan; 9 Department of Radiation Oncology, Kaohsiung Medical University Hospital, Kaohsiung Medical University, Kaohsiung, Taiwan; 10 Department of Surgery, Faculty of Medicine, College of Medicine, Kaohsiung Medical University, Kaohsiung, Taiwan; 11 Institute of Clinical Medicine, College of Medicine, Kaohsiung Medical University, Kaohsiung, Taiwan; 12 Department of Medical Research, Kaohsiung Medical University Hospital, Kaohsiung, Taiwan; University of Barcelona, Spain

## Abstract

**Background:**

The recurrence of colorectal cancer (CRC) is frequent within the first year of curative resection surgery and may be unavoidable. microRNAs have been suggested to play roles in carcinogenesis and cancer recurrence. We recently identified microRNA-29c **(**miRNA-29c**)** as a predictor of early recurrence in CRC. In the present study, we further investigated the functions and serum level of miRNA-29c in relation to early recurrence of CRC.

**Methods:**

First we further confirmed overexpression of miRNA-29c in non-early relapse subjects. Gain-of-function *in vitro* studies were used to evaluate the effect of miRNA-29c on cell proliferation, migration, invasion, and cell cycle progression. The colon cancer cell line Caco2 and a stable clone overexpressing miRNA-29c were xenografted to evaluate the *in vivo* effect of miRNA-29c in null mice. Finally, circulating miRNA-29c was investigated as a potential biomarker for identifying early relapse.

**Results:**

miRNA-29c expression significantly decreased during early relapse compared to non-early relapse in UICC stage II and III CRC patients (*P* = 0.021). *In vitro* studies showed that overexpression of miRNA-29c inhibited cell proliferation and migration. The cell cycle studies also revealed that miRNA-29c caused an accumulation of the G1 and G2 population. *In vivo*, miRNA-29c suppressed tumor growth in null mice. The serum miRNA-29c increased significantly in early relapsed patients compared to non-early elapsed patients (*P = *0.012).

**Conclusions:**

miRNA-29c shows anti-tumorigenesis activity, and preoperative circulating miRNA-29c levels can be used to predict postoperative early relapse of CRC.

## Introduction

The high incidence and mortality of colorectal cancer (CRC) pose a significant public health problem worldwide [1]. Although surgical resection can be highly effective for a localized disease, 30–40% of patients develop recurrence after surgery [2] and 40–50% of recurrences apparent within the first year after initial surgical resection [3], [4]. The time from the initial treatment to the development of recurrence has been reported to be strongly related to survival, particularly in patients within one year of their surgical resection [5]. Continuous efforts have been made to search for simple and reliable methods/biomarker for the early detection of tumors and for CRC prognosis in order to provide early, adequate, and effective treatment.

A mature microRNA (miRNA) is a small, noncoding RNA that contains approximately 20 nucleotides and can post-transcriptionally regulate the expression of several target genes at same time [6]. The tumorigenesis of CRC involves multi-step genomic changes, including the activation of oncogenes and inactivation of tumor suppressor genes [7]. miRNAs have been suggested to play roles in the development of cancers, including carcinogenesis, progression, and recurrence [7]–[10]. Most published studies have focused on tissue-based miRNA expression [8], [11], [12], and only a few studies have investigated circulating miRNAs in patients with CRC [13]–[15]. Recent studies have indicated that the serum levels of certain miRNAs can serve as potential biomarkers for various cancers [16]–[18].

We recently used a combination of bioinformatics and experimental approaches to predict and validate miRNA-29c as a candidate biomarker associated with CRC recurrence [19]. In the current study, we first used a large data set to confirm the relationship between miRNA-29c expression and early CRC recurrence. A series of *in vitro* and *in vivo* experiments were conducted to illustrate the functional role of miRNA-29c in CRC tumorigenesis. Subsequently, we explored whether the serum level of miRNA-29c can be used as a biomarker to predict early CRC recurrence.

## Methods

### Patients and Samples

To avoid the potential influence of neoadjuvant treatment on miRNA expression, patients were excluded if they had undergone neoadjuvant treatment with either chemotherapy or radiotherapy before surgery. The CRC tumor samples were obtained from 107 patients with primary CRC at UICC stages II/III (56 non-early relapsed patients and 51 early-relapsed patients after radical resection). Among these 107 patients, miRNA-29c data for 68 subjects (27 non-early relapsed patients and 41 early relapsed patients) have been reported in our pervious paper [19]. CRC early relapse was defined as local recurrence or distant metastasis that occurs within 1 year after radical resection [5], [20], [21] and the patients that relapsed after the first year and did not relapse during the time of the study were classified into the non-early relapsed group. Because the CRC recurrence rate in stage I is relatively low, and patients with stage IV CRC already have metastatic entities [19], [20], the present study only recruited CRC patients at stage II and stage III. CRC tissues were quickly frozen in liquid nitrogen after surgical resection. The serum samples from 61 CRC patients with primary CRC at UICC stages II/III (41 non-early relapsed and 20 early relapsed patients) before they received surgery were collected and serum miRNA-29c levels were measured. Serum miRNA-29c levels were also measured in 23 healthy subjects (14 female and 9 male). Both serum and CRC tumor samples were obtained from 38 subjects in this study. All subjects were unrelated ethnic Chinese residing in Taiwan. Written informed consent was obtained from each subject to collect their clinical samples and to publish these case details and all patient data were anonymized. The study protocol was approved by the Kaohsiung Medical University Hospital Institutional Review Board (Protocol Number: KMUH-IRB-990343).

### RNA extractions from Tissue

Approximately 100 mg of each tissue were homogenized using a bench-top homogeniser (Polytron PT1600E, Kinematica AG, Lucerne, Switzerland) and total RNA, including mRNA and miRNA, was purified using a Qiagen RNAeasy system (Qiagen, Hamburg, *Germany*), according to the manufacturer’s instructions.

### miRNA-29c Expression Levels in Early and Non-early Relapsed CRC Patients

To measure miRNA-29c expression levels, miRNA-29c cDNA was synthesized from 20 ng of total RNA with a unique primer (Applied Biosystems Inc., CA) and TaqMan miRNA RT-qPCR assay (Applied Biosystems Inc.) was used. The relative expression levels of miRNA-29c in tissues were normalized to that of U6b as an internal control using the equation: log_10_ (2^−ΔCt^), where ΔCt = (Ct_miRNA-29c_ - Ct_U6b_). The mean and standard deviation (SD) values of log_10_ (2^−ΔCt^) were calculated.

### Cell Culture

The human colon carcinoma cell line Caco2, Lovo, SW480 and SW620 (ATCC, Manassas, VA) was cultured in DMEM (Gibco-BRL, Gaithersburg, MD, USA) supplemented with 10% fetal calf serum (FCS; Gibco-BRL) and 100 U/mL of penicillin [21]. The cells were maintained at 37°C in an atmosphere of 5% CO_2_.

### Construction of a Plasmid for the Over-expression of miRNA-29c

The pCDH vector (System Biosciences, Mountain View, CA) was used to assess the functional consequences of miRNA-29c overexpression. We constructed the pCDH- miRNA-29c plasmid by inserting the miRNA-29c PCR product into the multiple cloning site regions of pCDH. The sequences of the primers used to amplify miRNA-29c were tcctgaattcgaggatgccctggagtattcgg and ctaggcggccgcgcatgatcttccttccctattc. The forward primer was extended at the 5′ end to include the gaattc sequence to create an *EcoR1* restriction site, and the reverse primer was elongated at the 5′ end to include the gcggccgc sequence to create a *Not1* restriction site. The construct was confirmed by direct DNA sequencing.

### Establishment of Stable Clones

Caco2 cells (5×10^5^) were seeded and transfected with 400 ng of the constructs (either the negative scrambled pCDH vector or the pCDH-miRNA-29c plasmid) using Lipofectamine 2000 (Invitrogen). The stably transfected Caco2 cells containing the pCDH-NC plasmid (NC: negative control) or the pCDH-miRNA-29c plasmid (O miRNA-29c: overexpression of miRNA-29c) were selected over a 4-week period by using standard culture medium supplemented with 12 µg/mL puromycin (Sigma-Aldrich Co, St. Louis, MI). Stable transfection of the plasmids was confirmed by performing a miR qRT-PCR assay.

### Analysis of Cell Proliferation

Caco2 stable clones cells were seeded and incubated for 22 h, then further incubated for another 2 h with 1/10 volume of WST-1 reagent (Roche Diagnostics, Corp., Indianapolis, IN) before the absorbance at 450 nm was quantified using a spectrophotometer [21].

To further examine the role of miRNA-29c in proliferation, we evaluated the effect of miRNA-29c over-expression on the growth of 3 different cancer cell lines (Lovo, SW480 and SW620). We transition transfected has-miRNA-29c miRNA (miR-29c mimic, Ambion, USA) or negative control (NC, mirVana miRNA mimic, Ambion, USA) into Lovo, SW480 and SW620 cells. Cells were seeded, incubated for 22 h, and then further analyzed as pervious described.

### Cell Cycle Analysis

After incubation for 36 h, cell cycle progression was quantified by propidium iodide (PI, Sigma-Aldrich Co.) staining and subsequent analysis on a FACScan cytofluorimeter (Becton Dickinson, NJ) with CellQuest software (BD Biosciences), according to the manufacturer’s instructions.

### Analysis of Cell Migration

Cell migration was assessed using Transwell polycarbonate membrane inserts (Millipore, GmbH, Schwalbach, Germany) in 24-well plates. The cells (2×10^4^) were plated onto 24-well millicells and allowed to migrate for 24 h at 37°C. The inserts were rinsed in 1X PBS, and the cells were removed from the membranes with 1% trypsin in cell culture lysis reagent (Promega, Corp., Madison, WI, USA). Cell migration was assessed by quantifying the green fluorescence of the pCDH vector as described previously [21].

### Wound-healing Assay

After forming a monolayer, a wound was created in the cell sheet by manual scraping with a 200-µL micropipette tip. The culture medium was then replaced and the cells were incubated at 37°C. Wound closure was monitored and photographed at various time points (0, 24, and 48 h) under a microscope.

### Matrigel Invasion Assay

Cell invasion was assayed using 24-well Transwell permeable supports (BD Biosciences, San Jose, CA) with 8-µm pore polycarbonate membrane inserts. The cells (1×10^4^) were placed on the upper chamber and allowed to invade for 24 h. Cell invasion was assayed as described previously [21].

### In vivo Animal Studies

Four-week-old Balb/c nude mice (body weight: 12.6 to 15.6 g) were purchased from BioLasco Taiwan Co., Ltd (Taipei, Taiwan) and maintained in a specific pathogen-free environment (certificate no.: 26-99S029). At 6 weeks of age, each nude mouse was injected subcutaneously with 1×10^7^ Caco2 cells (either NC or OmiR-29c; *N* = 4 per transfected cell line) in the neck area. The tumor diameter was measured and the tumor volume (cm^3^) was calculated using the following formula: volume = (width × length × height)/2 [22]. Animals were sacrificed 3 weeks after injection of tumor cells, and the tumors were examined and counted immediately without prior fixation. Prior to sacrifice, a noninvasive imaging system (the Ultra Sensitive Molecular Imaging System Berthold NightOWL, Berthold Technology, Oak Ridge, TN) was used to track the cancer cells *in vivo* and to confirm that miRNA-29c overexpressing plasmids were still present in the cancer cells. This imaging strategy provides a noninvasive and ultrasensitive method for detecting the transfected plasmids that express GFP. The animal use protocols approved by the Institutional Animal Care and Use Committee of Kaohsiung Medical University (IACUC Approval No: 99052) in accord with the Guiding Principles with the Care and Use of Laboratory Animals.

### Serum Preparation, RNA Extraction and miRNA-29c Expression Levels

The venous blood was obtained before operation. The blood samples were centrifuged at 3000 rpm for 15 min and the serum was aliquoted into 1.7-mL Eppendorf tubes. In the absence of a well-documented stably expressed endogenous circulating miRNAs to serve as normalization controls, *C. elegans* synthetic *lin-4* miRNA (Cel-lin-4, Part Number: 4398988, Invitrogen) that was added to the serum preparation before RNA extraction was used as a normalization control as in earlier studies [23], [24]. For RNA isolation from serum, 300 µL of serum were homogenized in 900 µL of Trizol LS according to the instructions of the manufacturer (Invitrogen) with small modifications: 6 µL of 1 nM Cel-lin-4 were added into serum samples. Then, 250 µL of chloroform was added to the sample and the mixed solution was centrifuged. After an additional chloroform extraction and precipitation with isopropanol, the pellet was washed twice by centrifugation with 70% ethanol. The RNA pellet was dried for 10 min at room temperature and dissolved in 30 µL of distilled water. DNase treatment (Qiagen) was carried out to remove any contaminating DNA.

To measure miRNA-29c expression levels, miRNA-29c cDNA was synthesized and the TaqMan miR RT-qPCR assay was preformed as pervious described. The relative expression levels of miRNA-29c in serum were normalized to that of Cel-lin-4 using the equation: log_10_ (2^−ΔCt^), where ΔCt = (Ct_miRNA-29c_ - Ct_Cel-lin-4_). The mean and standard deviation (SD) values of log_10_ (2^−ΔCt^) were calculated.

### Statistical Analysis

The continuous variables were presented as mean ± standard deviation (SD) values, and the dichotomous variables were analyzed by Pearson’s chi-squared test and presented as number and percentage values. Analysis of covariance was performed using the JMP software (version 7.0.1, SAS Institute Inc., Cary, NC) to compare the mean miRNA-29c levels between early and non-early relapsed CRC patients. Age, sex, and stage of CRC were included as covariables in the statistical models. A 2-tailed *P* value of less than 0.05 was considered statistically significant. The statistical power was calculated using the online DSS Research Statistical Power Calculator (http://www.dssresearch.com/Home.aspx), on the following basis: one-tailed test, an alpha of 0.05, serum of 20 early relapsed patients and 41 non-early relapsed patients, and the empirical expression levels in each group.

## Results

### Demographic Data

The characteristics of the 107 independent CRC patients (56 non-early relapsed and 51 early relapsed patients) are summarized in Table S1. The mean ages (years) of the early relapsed and non-early relapsed patients were 67.20 and 64.90, respectively, with the range being 24 to 88 years. Information regarding the disease stage, sex, and age of the early relapsed and non-early relapsed patients is shown in Table 1. Early relapse was significantly associated with tumor type and perineural invasion (*P*<0.05, Table 2), but age, sex, tumor size, UICC stage of patients or histology distributions were not significantly different between early and non-early relapsed patients (*P*>0.05, Table 2).

**Table 1:** Demographic data in 107 UICC^1^ stage II-III colorectal cancer patients.

Variables	Number (%)
Age (y/o)	
<65/≧65	41(38.32)/66(61.68)
Gender	
Male/Female	66(61.68)/41(38.32)
Tumor size (cm)	
<5/≧5	56(52.34)/51(47.66)
Tumor invasive depth	
T2/T3/T4	4(3.74)/91(85.05)/12(11.22)
Tumor stage	
II/III	57(53.27)//50(46.73)
Vascular invasion	
No/Yes	76(71.03)/31(28.97)
Perineural invasion	
No/Yes	79(73.83)/28(26.17)
Tumor grade	
WD/MD/PD^2^	1(0.94)/92(85.98)/14(13.08)
Tumor type	
Ac/M/S^3^	96(89.72)/10(9.35)/1(0.94)
Postoperative early relapse^4^	
No/Yes	56(52.34)/51(47.66)

1 UICC: Union for International Cancer Control.

2 WD:Well differentiated; MD: Moderately differentiated; PD: Poorly differentiated.

3 Ac: Adneocarcinoma; M: Mucinous carcinoma; S: Signet-ring cell carcinoma.

4 It means local recurrence or distant metastases within 12 months after surgery.

**Table 2:** Univariate and multivariate analysis of predictors on postoperative early relapse for 107 UICC^1^ stage II-III colorectal cancer patients undergoing curative resection.

	Non-early relapse	Early relapse	Univariate analysis	Multivariate analysis	
Parameters	(N = 56) (%)	(N = 51) (%)	*P* value	OR^2^ (95% CI^3^)	*P* value
Gender (Female/Male)	20/36	21/30	0.562	1.051(0.421–2.661 )	0.916
Age (<65/≧65)(y/o)	18/38	23/28	0.168	0.791(0.310–2.035 )	0.624
Size (<5/≧5)(cm)	32/24	24/27	0.297	1.397(0.536–3.702 )	0.494
Depth (/T2/T3+T4)	3/53	1/50	0.343	5.074(0.515–118.151)	0.170
Stage (II/III)	34/22	23/28	0.105	1.652(0.626–4.457)	0.311
Vascular invasion (No/Yes)	44/12	32/19	0.071	0.778(0.235–2.444 )	0.669
Perineural invasion (No/Yes)	48/8	31/20	0.003	4.516(1.442 –15.424)	0.009
Grade (PD/WD+MD^4^)	6/50	8/43	0.446	5.346(0.707 –63.816)	0.109
Type (M+S/Ac^5^)	2/54	9/42	0.014	0.053(0.003–0.504)	0.008
miRNA-29c expression^6^Mean±SD^7^	0.67±0.71	0.37±0.59	0.021	2.722 (1.301–6.172)	0.007

1 UICC: Union for International Cancer Control;

2 OR: Odds ratio;

3 CI: Confidence interval.

4 WD:Well differentiated; MD: Moderately differentiated; PD: Poorly differentiated.

5 Ac: Adneocarcinoma; M: Mucinous carcinoma; S: Signet-ring cell carcinoma.

6 The relative expression level of miRNA-29c is represented by log_10_ (2^−ΔCt^). ΔCt = (Ct_miRNA-29c_ - Ct_U6b_), with U6b as the internal control for normalization.

7 SD: Standard deviation.

We collected serum samples from 61 CRC patients (41 non-early relapsed and 20 early relapsed patients) before they received surgery. The characteristics of the 61 CRC patients for the serum study are summarized in Table S2. Of the 61 patients who donated preoperative serum samples, CRC tumor samples were also available for 38 patients (30 non-early relapsed and 8 early relapsed patients). Therefore, both serum and CRC samples were simultaneously analyzed for 38 patients in the present study.

### miRNA Expression in Early and Non-early Relapsed Patients

**Figure 1:**
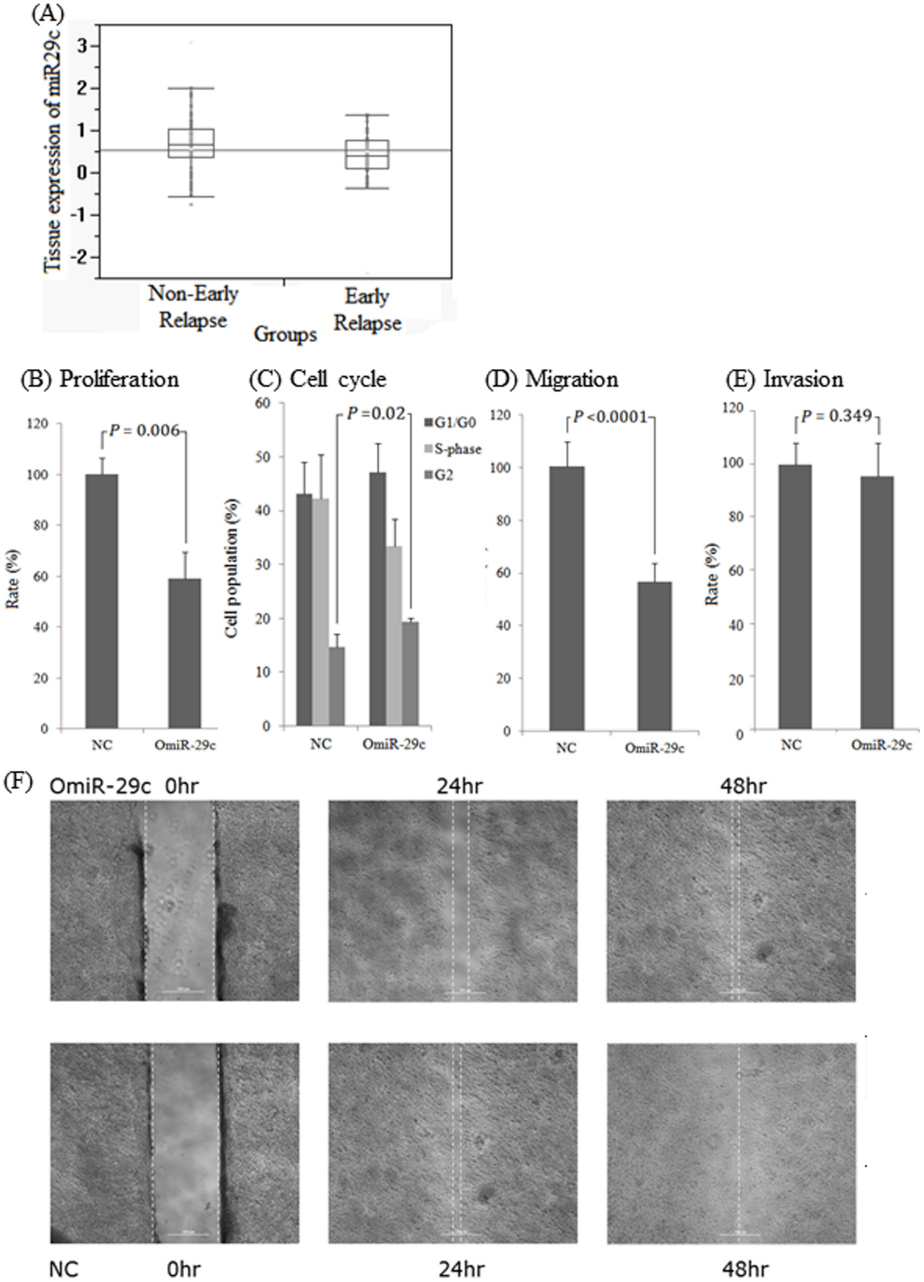
The expression levels of miRNA-29c are deregulated in human CRC tumors, and overexpression of miRNA-29c in Caco2 cells affects cell proliferation, cell cycle progression, and cell migration. (A) miRNA-29c expression levels in 56 non-early and 51 early relapse CRC tumor samples. The expression level is significantly decreased in the early-relapse tumors (*P = *0.021). (B) WST-1 assay showing that miRNA-29c suppresses tumor cell proliferation (*P = *0.006). OmiR-29c indicates the Caco2 cell clone stably overexpressing miRNA-29c, and NC indicates the negative control stable clone. (C) Overexpression of miRNA-29c causes a significant accumulation of cells in the G2 phase (*P = *0.02). Black: G1/G0 phase; light gray: S phase; and dark gray: G2 phase. (D) Transwell assay showing that overexpression of miRNA-29c suppresses tumor-cell migration (*P*<0.0001). (E) Overexpression of miRNA-29c does not affect tumor-cell invasion (*P = *0.349). (F) Overexpression of miRNA-29c decreases cell migration, as indicated by a gap of increased width in O miRNA-29c compared to NC at 24 and 48 h. The photographs showing the inhibition of tumor cell migration were obtained from the wounding healing assay.

The miRNA-29c expression levels in 107 CRC tumor samples were different between early and non-early relapsed patients. The mean of log_10_ (2^−ΔCt^) was 0.67 in the non-early relapsed group and 0.37 in the early relapsed group (*P = *0.021, Fig. 1A). Therefore, the miRNA-29c levels were lower in the samples from early relapsed patients by 2.00-fold compared to that in samples of non-early relapsed patients. This is similar to our previous report of a 2.04-fold change [19]. Early relapse was significantly associated with perineural invasion, tumor type and lower expression of tumor miRNA-29c by univariate and multivariate analyses (Table 2), but the miRNA-29c expression levels were not significantly associated with any clinicopathological variable (Table 3). Therefore, the expression of miRNA-29c is an independent predictor factor for detecting the early relapse of CRC. These results again verify our previous data [19], and support miRNA-29c as a potential predictor of the early relapse of CRC after surgery.

**Table 3:** miRNA-29c expression levels in colorectal cancer tumor samples.

Variables	mean ± SD	*P*
Sex (male/female)	0.60±0.63/0.41±0.73	0.161
Age (<65 yr/≥ 65 yr)	0.55±0.55/0.51±0.74	0.731
Maximum size (<5 cm/≥ 5 cm)	0.61±0.68/0.44±0.65	0.190
Location (colon/rectum)	0.51±0.69/0.56±0.63	0.726
UICC^1^ Stage (II/III)	0.61±0.75/0.43±0.56	0.180
Depth of invasion (T_2_/T_3_/T_4_)	0.06±0.51/0.56±0.69/0.46±0.55	0.323
Vascular invasion (no/yes)	0.54±0.72/0.48±0.55	0.642
Perineural invasion (no/yes)	0.53±0.71/0.52±0.57	0.931
Histology (WD/MD/PD^2^)	0.78/0.57±0.60/0.20±0.99	0.138
Type of tumor (A/M/S^3^)	0.53±0.69/0.50±0.57/0.46	0.988
Early relapse^4^ (no/yes)	0.67±0.71/0.37±0.59	0.021*

1 International Union Against Cancer.

2 WD: well differentiated; MD: moderately well differentiated; PD: poorly differentiated.

3 A: adenocarcinoma; M, mucinous; S: signet-ring cell carcinoma.

4 Early relapse means recurrence of cancer within 12 months after surgery.

* P value was considered statistically significant.

To clarify the role of miRNA-29c expression in early relapsed patients, we further compared miRNA-29c expression between relapsed *vs.* non-relapsed groups. Seven patients of the non-early relapsed group were relapsed after first year; hence, were re-classified into relapsed group. The miRNA-29c expression levels between relapsed and non-relapsed patients and the mean of log_10_ (2^−ΔCt^) was 0.65 in the non-relapsed group and 0.43 in the relapsed group (*P* = 0.099, Fig. not shown).

### Overexpression of miRNA-29c Influences Cell Proliferation and Cell Cycle Progression

To examine the role of miRNA-29c in tumorigenesis, we evaluated the effect of miRNA-29c on the growth of Caco2 cells. We established a Caco2 stable clone (OmiR-29c) overexpressing miRNA-29c. The mean miRNA-29c level in OmiR-29c was 0.45 (expressed as log_10_ (2^−ΔCt^)), which was higher than the mean level of miRNA-29c in the negative control (NC) clone by 2.82-fold. As shown in Fig. 1B, the proliferation rate of OmiR-29c was only 58.7% of the rate of NC after 24 h (*P = *0.006). Because some of the predicted miRNA-29c target genes are related to cell cycle progression and cell growth, we assessed whether miRNA-29c influenced the cell cycle by flow cytometry. Our data showed that OmiR-29c significantly increased the accumulation of the G2 cell population (19.4% in OmiR-29c *vs*. 14.7% in NC, *P = *0.02; Fig. 1C), slightly increased the accumulation of the G1/G0 population (47.1% in OmiR-29c *vs*. 43.1% in NC, *P = *0.43), and decreased the accumulation of the S-phase population (33.48% in OmiR-29c *vs*. 42.21% in NC, *P* = 0.14). These findings suggested that overexpression of miRNA-29c inhibits the growth of colon cancer cells due to the decreased S phase and inducing G2 arrest.

By proliferation analysis with transition transfected has-miRNA-29c miRNA (miRNA-29c mimic) or negative control (NC, mirVana miRNA mimic) into Lovo, SW480 and SW620 cells, we found that the proliferation rate of miRNA-29c over-expression cells were significantly down to 50.9% (Lovo) and 51.9% (SW480) of the rate of NC after 24 h (*P = *0.016 and 0.009, respectively, Fig. S1). However, the proliferation rate of SW620 was not shown significantly different (down to 92.4%) in the miRNA-29c over-expression cells. Consequently, in addition to CaCo2, miRNA-29c would also suppress the growth of colon cancer cell lines: Lovo and SW480, but not SW620.

### Effects of miRNA-29c Overexpression on Cell Migration

The data from the transwell assay (Fig. 1D) indicated that OmiR-29c displayed slower migration than did NC by 56% (*P*<0.0001). Inhibition of migration was also evaluated in a wound-healing assay in which the width of the wound was 800 µm. The widths of the gaps in NC and OmiR-29c were 100 and 180 µm at 24 h, respectively, and were 0 and 100 µm at 48 h, respectively. These findings suggested that cell migration was significantly reduced in OmiR-29c. These assays consistently showed that cell migration was dramatically decreased when miRNA-29c was overexpressed (Fig. 1F).

### Effects of miRNA-29c Overexpression on Tumor Cell Invasion

The data from the Matrigel invasion assay showed no significant differences between the OmiR-29c and NC clones regarding their capacity for invasion. This finding suggested that overexpression of miRNA-29c did not affect invasion by the tumor cells (*P = *0.349, Fig. 1E).

### Effect of Overexpression of miRNA-29c in Nude Mice

**Figure 2:**
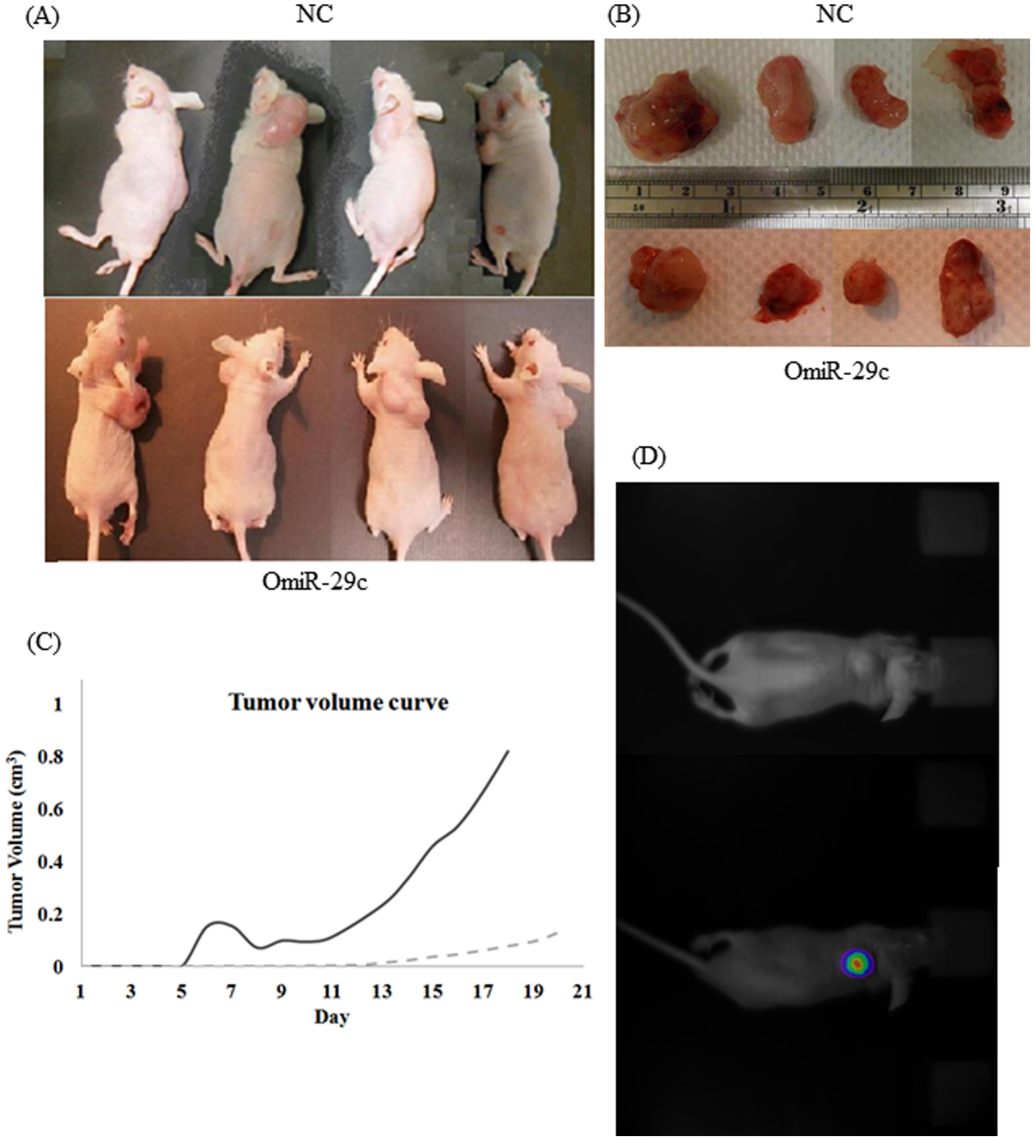
Effects of miRNA-29c on tumor growth in an animal model (*in vivo*). OmiR-29c is the Caco2 clone stably overexpressing miRNA-29c, and NC is the control stable clone. (A) Photographs of null mice subcutaneously injected with NC cells (*N = *4, upper panel) or with OmiR-29c cells (*N = *4, lower panel) were taken 21 days post-injection. (B) The tumor lumps were smaller in the OmiR-29c group (lower panel) than in the NC group (upper panel) after 21 days. (C) The tumor (cm^3^) growth curves for OmiR-29c (- - -; gray) and NC cells (–-; black) over 21 days (*P = *0.018). (D) Using the Ultra Sensitive Molecular Imaging Strategy, the luciferase activity in the lumps can be scanned without invasion from photographs of null mice subcutaneously injected with OmiR-29c cells taken 21 days post-injection.

To further validate the anti-tumorigenesis effect of miRNA-29c, we examined the effect of miRNA-29c overexpression on tumor growth *in vivo*. After subcutaneous injection of the OmiR-29c and NC clones in the neck of nude mice, the tumor masses became palpable 7 days after inoculation and were allowed to grow until the end of third week (Fig. 2A). Mice that received OmiR-29c cells had significantly smaller cancer lumps than did those that received NC cells (*P = *0.018; Fig. 2B and 2C). The data from Ultra Sensitive Molecular Imaging Strategy confirmed the overexpression of miRNA-29c in the OmiR-29c clone 3 weeks after seeding tumor cells via detection of green fluorescence *protein* (Fig. 2D). The results from the *in vivo* experiment provided further support the fact that overexpression of miRNA-29c results in a reduction of tumor proliferation in experimental animals.

### Circulating miRNA Expression in Early and Non-early Relapsed Patients

**Figure 3:**
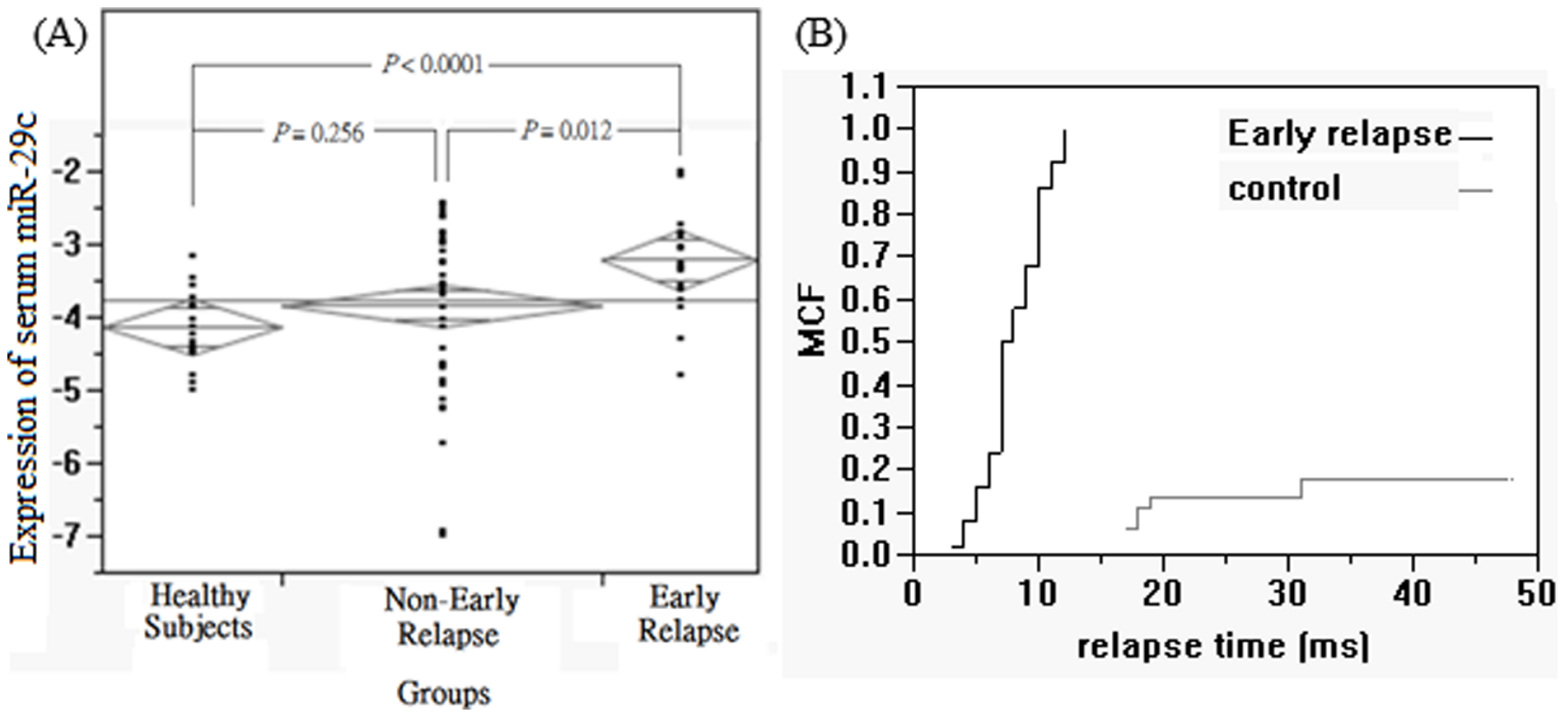
The expression levels of circulating miRNA-29c and recurrence analysis of the 107 patients with colorectal cancer (CRC). (A) The expression levels of circulating miRNA-29c are deregulated in the serum samples from 23 healthy subjects, 41 non-early and 20 early-relapse CRC patients. Serum miRNA-29c levels were not significantly different between healthy subjects group and non-early-relapse group (*P* = 0.256). The expression level is significantly increased in the serum samples of early-relapse patients comparing to the healthy subjects group (*P*<0.0001) and non-early-relapse group (*P = *0.012). (B) The recurrence analysis in CRC patients was assessed by the Kaplan-Meier method and the patterns of early relapsed (blank line) and non-early relapsed (gray line) groups were shown.

Preoperative serum miRNA-29c levels were significantly different between early and non-early relapsed patients. The mean of log_10_ (2^−ΔCt^) were −3.841 in the non-early-relapse group and −3.216 in the early-relapse group (Fig. 3A). Serum miRNA-29c levels were not significantly different between healthy subjects group and non-early-relapse group (*P* = 0.256), but the miRNA-29c increased significantly in early relapsed patients compared to the healthy subjects group and non-early relapsed group (*P*<0.0001 and *P = *0.012, respectively). Based on the serum data, our sample size of 61 achieved a statistical power of 83.1%. The recurrence analysis of early relapsed (blank line) and non-early relapsed (gray line) groups was assessed by the Kaplan-Meier method and the recurrence patterns were shown in Fig. 3B.

## Discussion

Increasing investigation into the function of miRNAs has created a new era in which tumorigenesis can be better understood. However, the role of miRNAs in the recurrence of CRC, particularly in early relapsed CRC, remains unclear. Recently, using computational analysis of mRNA expression profiles, we reported that miRNA-29c is a predictor of early relapsed CRC [19]. In the present study, we used an increased sample size to confirm that miRNA-29c expression in tumor tissue and serum can predict CRC early recurrence, and we conducted a series of *in vitro* and *in vivo* functional studies to further clarify the role of miRNA-29c in CRC tumorigenesis. *In vitro,* we showed that miRNA-29c prominently inhibits colon cancer cell proliferation and migration, but not invasion. Overexpression of miRNA-29c can arrest the cell cycle in the G2 phase, leading to the inhibition of cell proliferation. In nude mice, colon cancer cells overexpressing miRNA-29c displayed a significantly slower growth rate than did control cancer cells. Moreover, the preoperative serum levels of miRNA-29c were prominently associated with postoperative early recurrence. Accordingly, our results from cellular, animal, and human studies consistently indicated that miRNA-29c exerts an anti-tumorigenesis effect, and it may be a useful biomarker for the prediction of early relapse in CRC patients after the operation.

Recent studies have revealed that miRNA-29c is involved in a variety of biological processes, including carcinogenesis of various human cancers such as chronic lymphocytic leukemia [25], lung cancer [26], prostate cancer [27], and invasive breast cancer [28]. However, except our recent publication [19], studies related to the role of miRNA-29c in CRC pathogenesis and recurrence are limited. The critical role of miRNA-29c in the regulation of gene expression in tumorigenesis remains to be explored via targeted mRNA profiling. Tumor necrosis factor alpha-induced protein 3 (TNFAIP3), a key regulator in inflammation and immunity, was found to be inversely correlated with miRNA-29c levels and was identified as a target of miRNA-29c [29]. Overexpression of miRNA-29c in hepatitis B virus-related hepatocellular carcinoma cells effectively suppressed TNFAIP3 expression and HBV DNA replication, as well as inhibiting cell proliferation and inducing apoptosis [29]. miRNA-29c can affect tumorigenesis by working within the confines of well-known tumor suppressor (i.e., p53) pathways. The tumor suppressor p53, encoded by the *TP53* gene, is recognized as the guardian of the human genome because it regulates many downstream genes to exercise its function in cell cycle progression and cell death. Kumar *et al.* pointed out that the human *TP53* gene can be negatively regulated by several miRNAs: miRNA-125b, miRNA-504, miRNA-25, and miRNA-30d [30]. A recent study has shown that miRNA-29 is also involved in the p53 pathway, and miRNA-29 family members (miRNA-29a, miRNA-29b, and miRNA-29c) can upregulate p53 levels and induce apoptosis in a p53-dependent manner by directly suppressing p85 alpha (the regulatory subunit of PI3 kinase) and CDC42 (a Rho family GTPase [31]. Our recent work on miRNA-29c target genes suggested that DNMT3A, DNMT3B, and p53 are probably involved in the potential pathway of CRC development [19]. The above studies provide evidence for the role of miRNA-29c in suppressing CRC progression or recurrence. Likewise, a recent study indicated that miRNA-29c was frequently downregulated in tissues affected by esophageal squamous cell carcinoma, and miRNA-29c could suppress tumor growth by inducing cell cycle G(1)/G(0) arrest mainly through the modulation of cyclin E expression [32]. Similarly, we demonstrated that overexpression of miRNA-29c can inhibit the proliferation of colon cancer cells by reducing the accumulation of the S phase population.

Our data indicate that miRNA-29c prominently inhibits the proliferation and migration of colon cancer cells, but has no effect on cell invasion. Previously, miRNA-29c was reported to affect the motility and migration of nasopharyngeal carcinomas cells by inhibiting the expression of extracellular matrix proteins [33]. In contrast, altered cell motility and migration in esophageal squamous cell carcinoma cells transfected with miRNA-29c precursor molecules was not observed [32]. Downregulated miRNA-29c is involved in tumorigenesis and in controlling the loss of cell cycle progression in esophageal squamous cell carcinoma [32], for which the results are similar to our current findings in CRC. These above results reveal that deregulated miRNA-29c expression may play different roles in various cancers. In this study, the mean miRNA-29c level was 2.00-fold lower in the tumors of early relapsed patients compared to non-early relapsed patients. Moreover, the mean expression levels of miRNA-29c in tumor tissues were not significantly different between relapsed and non-relapsed patients (*P* = 0.099); hence, the prominently differences of miRNA-29c expression levels only in early *vs*. non-early relapsed groups further emphasize its role of detecting early relapse.

Serum miRNA-29c levels were higher by 4.22-fold in the samples of early relapsed patients compared to that in the samples of non-early relapsed patients. We showed that a low tumor level of miRNA-29c but a high serum level of miRNA-29c was associated with CRC early relapse. Although these results appear contradictory, similar findings have been reported for other cancers and other miRNAs recently. For example, miRNA-29c expression was significantly downregulated in tumor tissues from lung cancer patients with poor prognosis [34], while serum miRNA-29c levels were increased in early-stage non-small cell lung cancer compared to normal subjects [23]. Notably, the reverse pattern between serum and tissue miRNA-29c levels only remained if we analyzed the 38 patients in whom both serum and tissue miRNA-29c levels were determined simultaneously (data not shown). The contradictory miRNA expression levels in tumor tissues and serum have been also demonstrated for miRNA-1, miRNA-106, miRNA-146b, miRNA-155, miRNA-17-5p, and miRNA-27 (supplementary table 1 of [23]), and this phenomenon remains unexplained and needs to be explored further. Given that miRNA-29c mediates an anti-tumorigenesis effect, the higher serum level in the patients with the poorest prognosis may reflect a natural protective/defense mechanism, whereby the extra colon tissues express more miRNA-29c to reduce the tumor load.

In summary, the present data show that miRNA-29c mediates anti-oncogenic effects, including the inhibition of colon cancer cell proliferation and migration, and tumor growth, *in vivo* and *in vitro*. The association between decreased levels of miRNA-29c in tissues and increased levels of circulating miRNA-29c in serum with early relapse in UICC stage II and III CRC patients indicates that miRNA-29c is a potential biomarker for identifying high-risk CRC patients after radical resection. Therefore, enhanced surveillance or even an intensive therapeutic program may be needed for these patients.

## Supporting Information

Figure S1 Overexpression of miRNA-29c in Lovo, SW480 and SW620 cells affects cell proliferation. NC (black) indicates the transition transfected negative control clone and miRNA-29c (gray) indicates the transition transfected overexpressing miRNA-29c cells. By WST-1 assay, the result shown that miRNA-29c suppresses Lovo and SW480 cells proliferation (*P* = 0.016 and 0.009, respectively) but not SW620 cells (*P* = 0.415).
(TIF)

Table S1 (DOCX)

Table S2 (DOCX)
