# Supplementary figures and images for: The Functional Significance of MicroRNA-29c in Patients with Colorectal Cancer: A Potential Circulating Biomarker for Predicting Early Relapse
Source: PLoS One. 2013 Jun 28;8(6):e66842. doi: 10.1371/journal.pone.0066842 (PMC3696003; doi:10.1371/journal.pone.0066842)

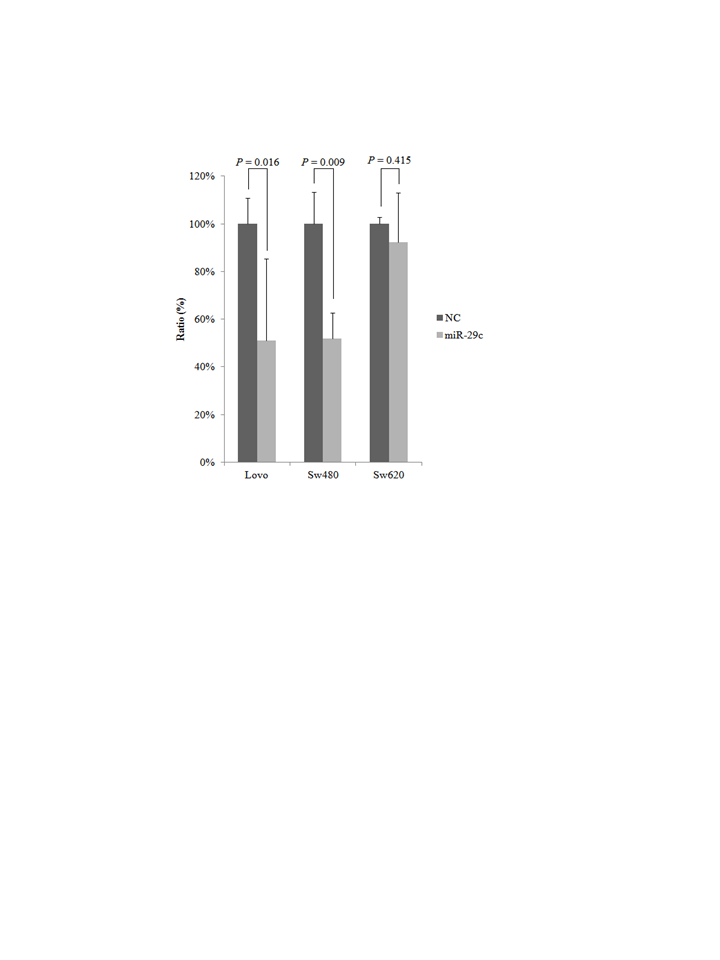

Supplement: Figure S1 — Overexpression of miRNA-29c in Lovo, SW480 and SW620 cells affects cell proliferation. NC (black) indicates the transition transfected negative control clone and miRNA-29c (gray) indicates the transition transfected overexpressing miRNA-29c cells. By WST-1 assay, the result shown that miRNA-29c suppresses Lovo and SW480 cells proliferation (P = 0.016 and 0.009, respectively) but not SW620 cells (P = 0.415). (TIF) [file pone.0066842.s001.tif]
