# Supplementary material for: The Functional Significance of MicroRNA-29c in Patients with Colorectal Cancer: A Potential Circulating Biomarker for Predicting Early Relapse
Source: PLoS One. 2013 Jun 28;8(6):e66842. doi: 10.1371/journal.pone.0066842 (PMC3696003; doi:10.1371/journal.pone.0066842)
Supplement: Table S1 — (DOCX) [file pone.0066842.s002.docx]

**Table S1.** Clinicopathological characteristics of the 107 UICC^a^ stage II/III colorectal cancer patients included as cohort I in the present study

| **Variables** | **Number (%)** |
| --- | --- |
| Sex (male/female) | 66 (61.68)/41 (38.32) |
| Age (<65 yr/≥65 yr) | 41 (38.32)/66 (61.68) |
| Maximum size (<5 cm/≥5 cm) | 56 (52.33)/51 (47.66) |
| Location (colon/rectum) | 78 (72.90)/29 (27.10) |
| Stage (II/III) | 57 (53.27)/50 (46.73) |
| Depth of invasion (T_1_/T_2_/T_3_/T_4_) | 0/4 (3.74)/91 (85.05)/12 (11.22) |
| Vascular invasion (no/yes) | 76 (71.03)/31 (28.97) |
| Perineural invasion (no/yes) | 79 (73.83)/27 (26.17) |
| Histology (WD/MD/PD^b^) | 1 (0.94)/92 (85.98)/ 14 (13.08) |
| Type of tumor (A/M/S^c^) | 96 (89.72)/10 (9.35) /1 (0.94) |
| Early relapse^d^ (no/yes) | 56 (52.34)/51 (47.66) |

^a^International Union Against Cancer

^b^WD: well differentiated; MD: moderately well differentiated; PD: poorly differentiated

^c^A: adenocarcinoma; M: mucinous carcinoma; S: signet-ring cell carcinoma

^d^Early relapse means recurrence of cancer within 12 months after surgery
