# Supplementary material for: The Functional Significance of MicroRNA-29c in Patients with Colorectal Cancer: A Potential Circulating Biomarker for Predicting Early Relapse
Source: PLoS One. 2013 Jun 28;8(6):e66842. doi: 10.1371/journal.pone.0066842 (PMC3696003; doi:10.1371/journal.pone.0066842)
Supplement: Table S2 — (DOCX) [file pone.0066842.s003.docx]

**Table S2.** Clinicopathological characteristics of the 61 UICC^a^ stage II-III colorectal cancer patients included as cohort II in the serum study

| **Variables** | **Number (%)** |
| --- | --- |
| Sex (male/female) | 41 (67.21)/20 (32.79) |
| Age (<65 yr/≥65 yr) | 25 (40.98)/36 (59.02) |
| Maximum size (<5 cm/≥5 cm) | 32 (52.46)/29 (47.54) |
| Location (colon/rectum) | 54 (88.53)/7 (11.48) |
| UICC^a^ Stage (II/III) | 19 (31.15)/42 (68.85) |
| Depth of invasion (T_1_/T_2_/T_3_/T_4_) | 2 (3.28)/1 (1.64)/46 (75.41)/12 (19.67) |
| Vascular invasion (no/yes) | 47 (77.05)/14 (22.95) |
| Perineural invasion (no/yes) | 45 (73.77)/16 (26.23) |
| Histology (WD/MD/PD^b^) | 2 (3.28)/54 (88.53)/5 (8.20) |
| Early relapse^c^ (no/yes) | 41 (67.21)/20 (32.79) |

^a^International Union Against Cancer

^b^WD: well differentiated; MD: moderately well differentiated; PD: poorly differentiated

^c^Early relapse means recurrence of cancer within 12 months after surgery
